# Legume Crops and Biotrophic Pathogen Interactions: A Continuous Cross-Talk of a Multilayered Array of Defense Mechanisms

**DOI:** 10.3390/plants9111460

**Published:** 2020-10-29

**Authors:** Davide Martins, Susana de Sousa Araújo, Diego Rubiales, Maria Carlota Vaz Patto

**Affiliations:** 1 Instituto de Tecnologia Química e Biologia António Xavier, Universidade Nova de Lisboa, Avenida da República, Estação Agronómica Nacional, 2780-157 Oeiras, Portugal; susana.araujo@blc3.pt (S.d.S.A.); cpatto@itqb.unl.pt (M.C.V.P.); 2 Association BLC3—Technology and Innovation Campus, Centre Bio R&D Unit, Rua Nossa Senhora da Conceição, 2, Lagares, 3405-155 Oliveira do Hospital, Portugal; 3 Instituto de Agricultura Sostenible, Consejo Superior de Investigaciones Científicas, Avenida Menéndez Pidal s/n, 14004 Córdoba, Spain; diego.rubiales@ias.csic.es

**Keywords:** legumes, airborne biotrophic pathogens, resistance mechanisms, powdery mildew, rust

## Abstract

Legume species are recognized for their nutritional benefits and contribution to the sustainability of agricultural systems. However, their production is threatened by biotic constraints with devastating impacts on crop yield. A deep understanding of the molecular and genetic architecture of resistance sources culminating in immunity is critical to assist new biotechnological approaches for plant protection. In this review, the current knowledge regarding the major plant immune system components of grain and forage legumes challenged with obligate airborne biotrophic fungi will be comprehensively evaluated and discussed while identifying future directions of research. To achieve this, we will address the multi-layered defense strategies deployed by legume crops at the biochemical, molecular, and physiological levels, leading to rapid pathogen recognition and carrying the necessary information to sub-cellular components, on-setting a dynamic and organized defense. Emphasis will be given to recent approaches such as the identification of critical components of host decentralized immune response negatively regulated by pathogens while targeting the loss-of-function of susceptibility genes. We conclude that advances in gene expression analysis in both host and pathogen, protocols for effectoromics pipelines, and high-throughput disease phenomics platforms are rapidly leading to a deeper understanding of the intricate host-pathogen interaction, crucial for efficient disease resistance breeding initiatives.

## 1. Introduction

Grain and forage legume species comprise the largest source of plant-based proteins, both for human and livestock consumption, especially in poorer areas where meat, fish, and dairy are economically inaccessible [1]. Their innate ability to form symbiotic associations with specific soilborne bacteria allows for atmospheric nitrogen fixation and conversion into a usable form, improving soil fertility and decreasing the demand for nitrogen inputs [2,3].

Approximately a tenth of all the fungi that have been described to date are capable of, to some extent, infect a vast array of crop species [4]. In the case of grain legumes, the impact of biotic threats can cause an estimated yield loss of 35–70% [5]. Despite having a significant impact on animal and human nutrition, studies on pathosystems including grain legumes have been relatively neglected when compared to other crops such as cereals. This highlights the urgent need to increase our knowledge of the defense responses in these crop species to successfully tackle the main biotic threats to their production. 

Biotrophic pathogens are dependent on host-derived photoassimilates and have a deep and prolonged physiological interaction with their host. Foliar diseases caused by biotrophic airborne fungal pathogens on legume crops include powdery mildews (*Erysiphe* spp., *Podosphaera* spp.), downy mildews (*Peronospora* spp.), and rusts (*Uromyces* spp., *Phakopsora* spp., *Puccinia* spp.) [6,7].

Plant disease resistance can be categorized or assorted in several concepts that are related to different aspects of their often overlapped nature, including the genetic background of the resistance (monogenic/polygenic), the effect on the measurable phenotypic response (complete resistance/partial resistance) or the effective broad-range or species-specific nature of resistance against the pathogen [8,9,10,11]. One important aspect while breeding for elite varieties relates to the expected resistance durability and stability once released to the agroecosystems. Traditionally, efforts to breed for disease resistance relied mainly on mechanisms controlled by major resistance (*R*) genes, since it is more amenable (due to their simpler genetic control) to be introduced into susceptible crops [12]. This approach leads to a dangerous scenario in which the nearly complete host resistance, allied to the mostly monogenic resistance promotes a rapid evolution of the pathogen and the appearance of more aggressive strains capable to overcome *R* gene-mediated defenses [13,14].

An intense bidirectional exchange of signals between the host plant and the biotroph takes place since spore deposition on the host surface until late stages of the infection process. The initial stages of pathogen development on the host plant do not differ greatly among biotrophs. Briefly, in response to the recognition of particular clues from the host surface, pathogenesis starts with spore germination, development of a germ tube towards suitable penetration sites, and appressoria differentiation from which the biotroph will attempt to gain access to host tissues. In powdery mildew pathogens penetration attempts will be made at the epidermal cell directly beneath the appressorium [15]. As for the majority of rust pathogens, germ tube development must be directed towards a stomata entrance, in which an appressorium will be formed and penetrate the stomata complex. Once inside the substomatal cavity, the pathogen will develop a substomatal vesicle (SSV), from which a hyphae emerges and attempt to penetrate the mesophyll cells [16]. Consequently, plants will perceive the physical and chemical pressure imposed on the cuticle and cell-wall and respond with inducible pre-penetration defense responses. At this stage, surface-localized pattern recognition receptors (PRR) can recognize pathogen-associated molecular patterns (PAMPs) that are conserved molecular signatures characteristic to a whole class of microbes; or damage-associated molecular patterns (DAMP) released from pathogen-induced alterations on the host cell [17]. This direct recognition of pathogen invasion triggers a wave of inducible defensive mechanisms (PAMP-triggered immunity, PTI) which includes, among others discussed in this review, the secretion of cell-wall-degrading enzyme inhibitors and hydrolytic enzymes against the pathogen (e.g., chitinases, glucanases, proteases); the accumulation of secondary metabolites toxic for the pathogen; or cell wall reinforcements (papillae). In the case of pathogens capable to overcome this layer immunity, an haustorium will be developed inside the host cell, and release a set of effectors targeted to specific subcellular compartments where they can subvert PTI [18,19]. In response to this, plants will present a second line of receptors known as resistance (R) proteins. R proteins (often belonging to the nucleotide-binding site and leucine-rich repeats-containing family, NLR) will directly or indirectly interact with specific effectors [12]. The recognition of these proteins induces a second, more rapid and specific (than PTI) layer of defense, termed effector-triggered immunity (ETI), frequently resulting in programmed cell death of already invaded host cells, a mechanism known to act at the post-penetration level and known as the hypersensitive response (HR) [12].

Despite the differences and the particular features of the two-layered defense briefly described above, PTI and ETI pathways should not be considered as discrete responses, and rather as a continuum resulting in the activation of an overlapping set of immune reactions. In line with this, numerous examples of pathogen-derived molecules that fall within the grey area of PAMP and effector concepts have been extensively discussed in previous reviews to which the reader is kindly directed to (Thomma et al. [20]). Despite this, the present review will continue to refer to the concepts as PTI and ETI; however, acknowledging that the plant innate immune system is largely controlled by encoded receptors that allow the perception of specific molecules cueing for the presence of a pathogen, and that multiple receptor-ligand interactions are taking place simultaneously.

A better understanding of the complex communication between plant and pathogen is of extreme importance when designing new approaches for plant protection. Such understanding can only be achieved through a multidisciplinary characterization of the many layers of plant immune response, deciphered at the cellular, genetic and molecular level. Our effort in the present review, to synthesize observations from different methodologies, will provide a holistic overview of the legume airborne biotrophic pathogen interaction, elucidating the major events that lead to effective defense response. This work, while highlighting where a lack of knowledge of specific components of defense responses still exists and discussing the significant advances in phytopathogen-oriented studies in legume crops, will identify new directions for future research.

## 2. Detangling the Multi-Layered Interaction between Legume Crops and Airborne Biotrophs

### 2.1. The Outer Layer—Cuticle as a Source of Physical and Chemical Clues for Pathogenicity

The cuticle, as the outer-most layer in plant tissues, plays many crucial roles in the interaction with biotic stresses. The most important aspects, regarding this layer, might be related to the more obvious physical barrier imposed on pathogen penetration but also to the myriad of chemical signals perceived by the microorganism triggering host specificity and the first steps of infection [21].

Spore deposition and adhesion to the leaf surface comprises the first and most crucial events in the establishment of the invading pathogen. Shortly after the deposition in the leaf surface, the release of adhesive extracellular material (ECM) from the spore, typical of many phytopathogenic fungi, provides a physical anchoring to the leaf surface, and potentially stimulate processes leading to spore germination [21,22]. In the case of *Erysiphe pisi* conidia, the causal agent for pea powdery mildew, ECM production, and secretion can be observed within 5 min after inoculation [22]. The composition of ECM consists on a vast array of chemical compounds, fungi species-specific, commonly containing glycoproteins, lipids, polysaccharides, cutinases, esterases, and other hydrolytic enzymes responsible for the enzymatic digestion of cuticle polymers to ease cell penetration [21,23]. 

After spore germination, appressorium differentiation and the following steps of pathogen development are strictly dependent on the recognition of particular morphological or chemical components of the host leaf surface. Gniwotta et al. [24] detected marked differences in the chemical composition and morphology of epicuticular waxes from adaxial and abaxial pea leave surfaces. Interestingly, the adaxial side of the leave, densely covered by wax crystals, proved to be more appropriate for *E. pisi* conidia germination and appressorium differentiation, comparing with the abaxial surface, with fewer wax crystals. Results from Fondevilla et al. [25] further highlighted that specific clues from epicuticular chemistry and structure could be important factors inducing pre-penetration resistance, observed as reduced conidia germination and appressorium differentiation in pea cultivars. In *Medicago truncatula*, loss-of-function mutation of *Inhibitor of Rust Germ tube differentiation1* (*IRG1*) induced major modifications in epicuticular wax content and composition on abaxial surfaces, compromising spore germination, differentiation of pre-infection structures, and host cell penetration of *Phakopsora pachyrhizi* (the direct-penetrating Asian soybean rust agent) and *Colletotrichum trifolii* (anthracnose agent) [26,27]. Further detailed cytological and chemical analysis showed that the abaxial leaf surface of *irg1* lacked epicuticular wax crystals and reduced surface hydrophobicity, compared with wild-type [27]. Consistent with the observed absence of wax crystals phenotype, the *IRG1* gene had sequence similarities with a Cys_2_His_2_ zinc finger transcription factor (TF), shown to control leaf development in *M. truncatula.* Additionally, *IRG1* loss-of-function mutation strongly down-regulated a key TF homolog of Arabidopsis (*MYB969*), involved in the regulation of wax biosynthesis [27,28].

Recognition of leaf wax composition and structure, as described above, is particularly important for rust pathogen development that relies on the location of stomata complexes to gain access to the plant tissue. Such preformed physical and chemical barriers are likely to contribute to non-host resistance to pathogens adapted to other plant families phylogenetically distant to the non-host [29]. On the non-host *Vicia faba* leaves infected with wheat stripe rust pathogen (*Puccinia striiformis* f.sp. *tritici*, *Pst*), germ tubes grew randomly on the leaf surface, thus resulting in a significant reduction in stomata location, as compared to *Pst*-infected wheat leaves [30]. Even in cases of germ tubes capable to locate and penetrate the stomata complexes, only 50% developed a normal substomatal vesicle (SSV), while the remaining contained aberrant SSV or did not have SSV structure. This observation might be due to marked differences in the topographic features and biochemical composition of epicuticular waxes in *V. faba* leaves compared to wheat. In contrast, in *M. truncatula* accessions infected with the adapted *U. striatus* (pathogens able to infect and develop appropriate infection structures and to overcome resistance barriers from the infected plant) and the non-adapted *U. fabae* and *U. lupinicolus* rust pathogens (pathogens less effective to overcome inducible or structural defense barriers), germ tubes of the three pathogens had a similar level of success in forming appressoria over stoma [31]. In this case, pre-invasive resistance mechanisms were of marginal importance in reducing infection severity.

### 2.2. From Spore Germination to Attempted Host Cell Invasion

Once the infection structures of biotrophic pathogen gain close contact with the host cell, attempts will be made to penetrate the cell wall and develop a haustorium inside the host cell to access the nutrient supply to support further host colonization. At this stage, inducible and/or constitutive defense reactions resulting in unsuccessful cell penetration by the pathogen are often referred to as pre-haustorial resistance.

Pre-haustorial resistance mechanisms are common in legume partial resistance against biotrophic pathogens. Examples are *Vicia faba* and lentil (*Lens culinaris*)-*U. fabae* [32,33]; pea-*U. pisi* [34] and *U. fabae* [35]; chickpea (*Cicer arietinum*)-*U. ciceris-arietini* [36]; *M. truncatula-U. striatus* [37], kudzu (*Pueraria* spp.)-*P. pachyrhizi* [38]; pea-*E. pisi* [39,40]. In most cases, resistance was manifested by a significant proportion of germinated spores that failed to form haustoria, either because the haustorial mother cells are not functional or due to a reduced chance to successfully differentiate a haustorium in the host cell, consequently hindering intercellular growth of the infection hyphae and growth of the colony [33,34,39]. In these examples, restriction to pathogen penetration is mainly attributed to the development of an localized cell wall reinforcement, or papillae, at sites of attempted penetration [35,40,41].

Microscopic observations and histochemical analysis allowed a better understanding of the composition and structure of cell wall strengthening and on how its arrangement culminates in a papillae-based resistance. Thickened cell wall and a dome-shaped papilla were observed in *V. faba* mesophyll cells in contact with the haustorial mother cell of the non-host wheat stripe rust pathogen (*Pst*) [30]. In this case, infection by *Pst* induced callose deposition in portions of the cell wall in direct contact with fungal structures, in papilla, and at encasement of haustoria. Coinciding with callose accumulation at 24 h after inoculation (HAI), transcription of *GSL5* (*Glucan Synthase-Like 5*), involved in callose formation, was up-regulated at 12–24 HAI. Similarly, while attempting to unveil the genetic control of *L. cicera* resistance to *E. pisi* through a linkage mapping approach, Santos et al. [42] identified Quantitative Trait Loci (QTL) that could contribute to the development of physical barriers. Through the comparative mapping of QTL intervals to the pea reference genome, the authors identified as candidate genes, the cellulose synthase family genes, which has been shown to contribute to the establishment of physical barriers by the deposition of cellulose in papilla [42,43].

Additional mechanisms restricting pathogen penetration were observed to confer resistance to *E. pisi*. Histological studies conducted by Iglesias-García et al. [40] on pea genotypes carrying the *er1* gene (the most widely deployed resistance gene in pea cultivars against powdery mildew) have shown that instead of callose apposition, penetration resistance was determined by cross-linking of host cell wall structural proteins (e.g., extensions and other glycoproteins rich in hydroxyproline,) [40]. Insolubilization of the mentioned cell wall proteins has been observed to occur in suspension-cultured common bean (*Phaseolus vulgaris*) cells within 5 min after exposed to glycan elicitors isolated from the cell walls of fungal pathogens. It was hypothesized that such rapid response could precede the establishment of other cell wall reinforcements, including callose deposition [44].

Lignin deposition further contributes to the penetration resistance conferred by the papillae, restricting and restraining the pathogen to the infection site. The expression of genes regulating phenylpropanoid and flavonoid pathways, involved in the production of phytoalexins and cell-wall reinforcing compounds such as lignins, has been reported to be strongly induced in soybean (*Glycine max*) cultivars upon infection with Asian soybean rust [45,46,47]. An initial peak in the expression of these genes was detected at early stages (12 HAI) in both resistant and susceptible soybean genotype; a second wave of gene expression took place later in the infection, which occurred at least 24 H earlier in the resistant soybean, as compared to the susceptible genotype [47]. Increased lignification of host tissues was also detected in partially resistant pea plants infected with *U. fabae* leading to a higher number of early aborted colonies and decreased colony size [35]. Virus-induced gene silencing (VIGS) of other enzymes involved in lignin biosynthesis (phenylalanine ammonia lyase, *GmPAL* or o-methyltransferases, *GmO-MT*) reduced lignin content by 30% and loss of resistance in soybean cultivars (carrying the resistance loci *Rpp2* and *Rpp1*) infected with *P. pachyrhizi* [48,49]. Nevertheless, it is worth mentioning that many secondary metabolites besides lignin, such as phytoalexins and other phenolic compounds that might express antifungal activity also derive from the phenylpropanoid pathway. Accordingly, it seems yet unclear which outcome of the phenylpropanoid pathway is the most significant to enhance resistance to biotrophic pathogens.

Complementing the establishment of cell wall reinforcements, the collapse of invaded host cells (hypersensitive response, HR) provides an additional layer of defense against biotrophic pathogens. Histological observations and characterization of *er2* and *Er3* powdery mildew resistance genes in pea showed a pronounced hypersensitive response after the formation of secondary haustoria at 72 HAI [50]. Similarly, in *Pisum fulvum* and several *M. truncatula* genotypes, the most effective resistance mechanism against *E. pisi* also involves a rapid and localized cell death of attacked epidermal cells, either as a rapid reaction against primary appressoria formation or as late-response following colony establishment [21,51,52] In kudzu-*P. pachyrhizi* early-acting HR, between 24–48 HAI, although initially observed in the penetrated epidermal cell, hypersensitivity extended to epidermal cells surrounding the penetrated epidermal cells and palisade mesophyll cells in proximity [38].

Microarray analyses of *E. pisi*-induced transcriptional changes on *M. truncatula* suggest that the onset of hypersensitive response might be partially regulated by salicylic acid (SA) via the EDS1 pathway [53]. In Arabidopsis, homologs to the up-regulated EDS1 in the *M. truncatula-E. pisi* pathosystem are essential components for the expression of *R*-gene based resistance via the SA-mediated EDS1/PAD4 pathway [54]. Additionally, homologs in *M. truncatula* of three common markers induced by the SA pathway known in Arabidopsis were up-regulated in response to *E. pisi* (thaumatin-like genes, *PR1, BGL2*) [53]. Evidences have been provided to the significance of other metabolic pathways modulating HR. Inhibition of enzymes involved in the phenylpropanoid pathway (e.g., cinnamyl alcohol dehydrogenase, CAD), previously described to contribute to cell-wall reinforcement, lead to a suppression of HR in *Vicia faba*-*U. fabae* and consequently increase of haustoria per colony and colony size [55].

### 2.3. Cytoskeleton Reorganization of Invaded Host Cells

Several studies have demonstrated the pivotal role of a rapid and localized reorganization of cytoskeleton components of host cells in close contact with phytopathogenic fungi [56]. The majority of cytoskeleton modification leads to the formation of cytoplasmic aggregates, nuclear and plastids movements, and rearrangements of endomembranes at pathogen contact sites [57,58,59]. Such reorganization of cytoplasmic content have been described by Chen and Heath [60] in an attempt to uncover the main cytological events culminating in hypersensitivity of cowpea leaf epidermal cells induced by cowpea rust pathogen (*Uromyces vignae*). Upon infection, the nucleus of invaded host cells migrates to the site of penetration, followed by Brownian motion of organelles and cytoplasm aggregation along the cell walls. Such modifications on cytoplasmic dynamics can be observed even before fungal penetration, ultimately controlling the onset of defensive strategies involved in penetration resistance and hypersensitive response [60]. 

**Figure 1:**
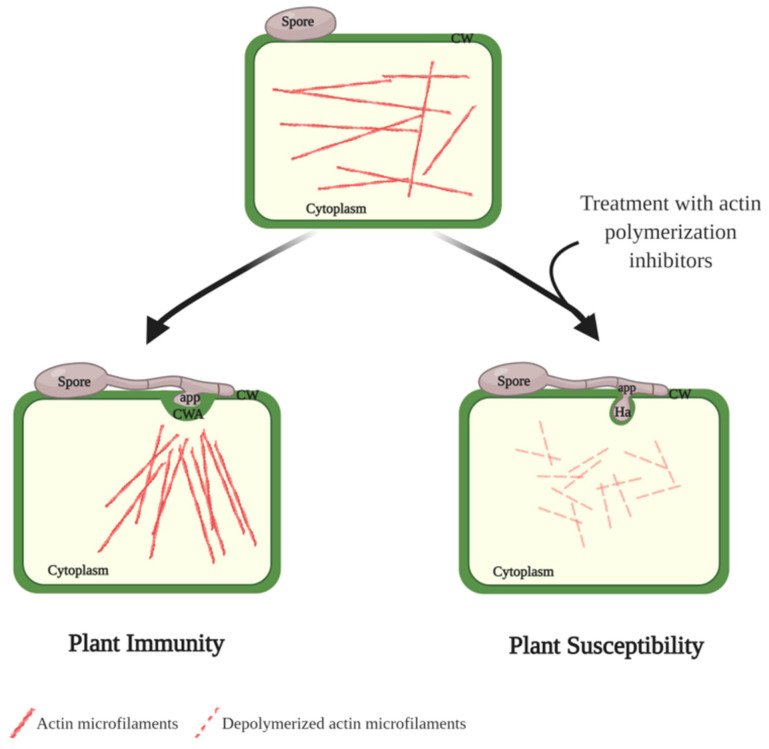
Reorganization of actin microfilament in response to pathogen infection. In non-attacked host cells, actin microfilaments form a homogenously dense and distributed network in the cytoplasm. Upon perception of attempted penetration by the pathogen, microfilaments increases in density and aggregates towards the penetration site. Actin focusing is required for rapid trafficking of cytoplasmic components, culminating in the establishment of cell-wall reinforcements. Treatment of leaves with inhibitors of actin polymerization has been shown to compromise cytoplasmic aggregation and suppress callose deposition and papillae formation, leading to increase susceptibility to pathogen infection. app, appressorium; CW, cell wall; CWA, cell wall apposition; Ha, haustorium (created with BioRender.com).

Actin microfilaments, and to some extent microtubules, are the main elements dictating cytoskeleton reorganization, by establishing a continuum of communication between the invading hyphae and the host plant nucleus, as well as other cytoplasmic components [61] (Figure 1). Indeed, following inoculation of pea plants with the non-adapted.

*Blumeria graminis* f. sp. *avenae* (*Bga*), confocal scanning microscopy images revealed an increase in the density of actin microfilaments focusing towards the site of contact between epidermal cells and appressorial germ tube [62]. Interestingly, when infected a second time with the adapted *E. pisi*, the previously observed rearrangements of cytoskeleton induced from a prior *Bga* inoculation were associated with induced inaccessibility to pea powdery mildew, measured as a significant reduction in the penetration efficiency. Treatment of *Bga* infected leaves with inhibitors of actin polymerization significantly reduced the induced inaccessibility of *Bga*/*E. pisi* co-infected epidermal cells, providing additional proof for the importance of actin-related defense mechanisms in limiting fungal penetration [62]. Similarly, cowpea (*V. unguiculata*) cultivars treated with inhibitors of actin microfilaments polymerization (cytochalasin E) lead to significant changes in the cytoplasmic reorganization of cells invaded by the cowpea rust fungi. In resistant cultivars, the treatment significantly reduced HR and deposition of callose in response to attempted penetration by the pathogen [63,64]. In contrast, the antimicrotubule agent oryzalin did not affect the establishment of penetration-related defenses in the interaction between *U. vignae* and the host and non-host cowpea and pea, respectively [63,65].

It is not yet clear whether these cytoskeleton responses are stimulated by fungal elicitors, by plant cell wall degradation products, or by physical pressure of appressorium adhesion or penetration peg emergence. Evidence was provided for the importance of the later, as abrasion of epidermal cells and treatment with hemicellulase induced nuclear migration in cowpeas, similar to what was observed when infected by rust fungi [57]. However, the observed actin rearrangement and altered cyclosis occurring before pathogen penetration hints for additional clues that could elicit cytoskeleton rearrangement. Overall, these studies suggest that the elements composing the cytoskeleton structure are critical factors in defense response against the invading pathogen, controlling a site-directed cytoplasmic stream and accumulation of defense-related compounds in the proximity of fungal penetration sites, needed for the onset of many protective strategies.

### 2.4. Reactive Oxygen Species Production and Oxidative Stress 

As evidenced by different studies, the oxidative burst is considered to be one of the most rapid defense mechanisms plants possess to cope with biotic stresses [66,67]. This response involves a strictly controlled accumulation of reactive oxygen species (ROS) primarily composed of hydrogen peroxide (H_2_O_2_) and superoxide (O_2_^-^) detected in host cells upon penetration by the pathogen and at sites of attempted penetration, in both compatible (successful infection leading to pathogen colonization and disease development) and incompatible (successful plant defense preventing pathogen establishment on host tissues) legume-pathogen interactions [68]

In many plant-microbe interactions, the characteristic oxidative burst can occur as a biphasic ROS accumulation. The first phase is an unspecific and shorter reaction occurring in both compatible and incompatible interactions [67]. The second long-sustained ROS burst, with higher magnitude, depends on the recognition of the pathogen’s avirulence (*Avr*) genes, assisting the establishment of disease resistance [67,68]. Given its membrane permeability and affinity to several plant signaling molecules as salicylic acid and nitric oxide (NO), the rapid production of reactive molecules like H_2_O_2_ can act as secondary messengers in defense-related signaling pathways, providing additional regulatory functions in defense responses [68,69]. Other reports have suggested that an oxidative burst can also contribute to the expression of pathogenesis-related genes and mediate the generation of phytoalexins and secondary metabolites [70,71]. In fact, ROS production in pea plants challenged with *E. pisi* was coupled with significant induction of phenylalanine ammonia lyase (PAL) enzyme [72]. Phenylalanine ammonia lyase, the first enzyme of the phenylpropanoid pathway, may provide infected cells with cinnamic acid, a precursor for lignin synthesis, and a variety of phytoalexins [71].

Other well-known defense mechanisms incited by ROS accumulation are the already mentioned localized collapse of infected host cells, termed hypersensitive response (HR), and the cell wall strengthening via oxidative cross-linking of structural proteins in the cell wall [67,68]. In resistant and partially resistant *M. truncatula* genotypes, the onset of a hypersensitive response after infection with *E. pisi* was consistent with the accumulation of hydrogen peroxide [53]. Resistant genotypes produced an early strong response at 12 HAI, in which 40% of conidia elicited H_2_O_2_ production, confirmed by 3,3-diaminobenzidine (DAB)-staining on whole-epidermal cell. Whereas with susceptible genotypes the proportion of conidia associated with staining never exceeded 9% [53].

The rapid and localized H_2_O_2_ accumulation after attempted penetration by the pathogen in the cowpea-*E. cichoracearum* and pea-*U. vignae* interactions seems to highlight its importance in the establishment of a physical barrier [66]. In fact, the treatment of cowpea and pea leaves with H_2_O_2_ scavenger catalase prior to inoculation with *E. cichoracearum* and *U. vignae*, respectively, resulted in increased fungal penetration efficiency. In contrast, treatment with the O_2_^-^ scavenger superoxide dismutase had no impact on fungal penetration efficiency [66]. Likewise, in the interaction between *V. faba* and the wheat stripe rust pathogen, H_2_O_2_ accumulation (but not O_2_^-^) was detected at the sites in direct contact with the substomatal vesicle (SSV) or haustorial mother cell and in papilla [30].

In plants, major ROS-scavenging mechanisms include catalase, ascorbate peroxidases, and superoxide dismutase enzymes [73]. The balance between the synthesis of reactive molecules and their removal by ROS-scavenging systems will strictly regulate the spatial-temporal accumulation of the generated oxidative burst in the host cell [64,70]. In cases in which the ROS-scavenging systems fail to contain oxidative burst, the excess of ROS accumulation leads to oxidative damage, promoting lipid peroxidation, damaging macromolecules such as pigments, proteins, nucleic acids, and lipids. Several studies have shown that resistant genotypes usually maintain malondialdehyde levels (MDA) constant through the course of infection [74,75]. Malondialdehyde is a known secondary end-product of lipid peroxidation, thus a proxy for cell membrane damage induced by the oxidative burst [74,75]. Accordingly, MDA levels in resistant pea cultivars were kept at significantly lower levels than in the susceptible, and negatively correlated to the activity of ROS-scavenging enzymes, including catalase and superoxide dismutase. Therefore, susceptible pea genotypes faced greater damage when infected with *E. pisi*, as they were less efficient in detoxifying ROS due to the low activity of antioxidant enzymes [75]. Ultimately, the modulation of antioxidative enzyme activity in response to pathogen attack is critical to maintain a steady-state level of ROS under tight control, preventing ROS-induced damage in the host cell, and promoting the ROS-dependent defense reactions [73].

Interestingly, pathogens have evolved mechanisms, through the secretion of different metabolites and enzymatic compounds, to prevent the oxidative burst and counteract the activation of ROS-induced resistance in the host plant [76]. One example of this was observed upon infection of *V. faba* with the rust *Uromyces fabae.* Voegele et al. [77] and Link et al. [78] provided evidence that mannitol and D-arabitol are released by *U. fabae,* and accumulates in the apoplastic fraction of the host *V. faba*. The concentrations at which these metabolites were detected in the apoplast were sufficient to effectively quench ROS, as observed in in vitro system, with a reduction of one-half when compared to the absence of mannitol [77,78].

## 3. Physiological Implications of Plant-Pathogen Interactions

### 3.1. Photosynthetic Performance in Attacked Legume Plants

Imaging systems provide significant insights on the extent of damage imposed by the pathogen on important physiological processes of the infected hosts, while mapping *in vivo* spatial-temporal changes in important components of the photosynthetic performance. Chlorophyll *a* fluorescence imaging yields a detailed analysis of the overall photosynthetic functions, giving indications on the light-dependent photosynthetic reactions, changes of photosynthetic metabolism, and indirectly estimates chlorophyll content in diseased leaves [79]. Chlorophyll *a* fluorescence imaging can also be applied as a rapid and non-destructive tool to follow changes in photosystem II (PSII) photochemistry, linear electron flux, and CO_2_ assimilation *in vivo* [80,81]. This is true for healthy leaves, as there is often a linear relation between the yield of PSII photochemistry and rates of CO_2_ assimilation [81]. However, given that in plants under biotic stress this linear relation is lost, it is beneficial to complement chlorophyll imaging analyses with gas exchange measurements [81]. 

Measurements with gas exchange systems provide valuable information on CO_2_ assimilation, transpiration rate, intercellular concentration of CO_2,_ and stomatal conductance [82,83]. The non-invasive nature of these techniques is particularly interesting while studying the impact of fungal infections as it allows for continuous measurement of the impact on main physiological processes throughout the infection development, and a better representation of the highly heterogeneous impacts on different regions of the infected leave.

The most studied physiological implications resulting from fungal pathogen infection relates to changes in basic physiological processes including dark respiration, photosynthetic activity, pigment concentration, transpiration rate, and altered translocation of photoassimilates [84,85,86]. The physiological impairments directly related to the photosynthetic apparatus can be in part explained by the destruction of portions of leaf cuticle by the pathogen’s enzymatic repertoire, disruption of stomatal movements, and reduction of air space in the stomatal chamber by the growing hyphae, compromising transpiration rate and gas exchange [87,88]. The effects of *U. appendiculatus* infection on common bean were described as inducing a decrease in the net carbon assimilation rate and an increase in dark respiration in diseased leaves, reaching a maximum throughout the sporulation phase [89]. Similar results were observed in *P. pachirhyzi* infected soybean plants [82]. The light absorption efficiency in soybean leaf photosynthesis decreased with increasing rust disease severity, in association with a reduction in chlorophyll content. A significant decline of optimal quantum yield of PSII (F_v_/F_m_) parameters pointed for a reduction in the efficiency of the electron transport rate of PSII and damage to the PSII reaction centers, indicating that a reduction in the CO_2_ exchange rate in infected leaves can in part be attributed to the quantum yield of the PSII photochemistry. 

*In vivo* chlorophyll *a* fluorescence imaging studies suggest that the impact on photosynthetic apparatus tend to be local and confined to leaf areas in close contact with infection structures and where symptoms, such as chlorotic and necrotic lesions, will develop. Parameters including minimal (F_0_) and maximal (F_m_) fluorescence and optimal quantum yield of PSII (F_v_/F_m_) were not significantly changed in apparently healthy regions of diseased leaves, as compared to those obtained in a leaf with no rust symptoms [84]. In common bean–*U. appendiculatus* pathosystems, Peterson and Aylor [90] detected regions with increased fluorescence emission in incipient lesions encircled by a halo of decreased emission affecting photosynthetic capacity in these areas. The radial increase in the size of the leaf tissue area with enhanced fluorescence emissions was consistent with the outward growth of the mycelium from the point of initial infection [90]. Additionally, this could be an indication that plants can regulate a localized decrease in photosynthetic activity and other assimilatory metabolic processes to induce respiration and other processes required to activate defense responses [84,87]. However, the complexity and heterogeneity of effects in photosynthetic apparatus between colonized and non-colonized tissue that can be detected in several pathosystems, makes it difficult to extrapolate and hypothesized generalized assumptions. 

Abnormal stomatal behavior is a common feature in plants exposed to pathogens, consequently interfering with photosynthesis, respiration, transpiration, and the ability to cope with subsequent stress events. In accordance, with increasing disease severity on common bean-*U. appendiculatus* pathosystem a reduced photosynthetic capacity and carbon assimilations rate was coupled with decreased stomatal conductance of infected leaf portions [89]. Interestingly, during disease development a slight increase on the ratio of intercellular CO_2_ to ambient CO_2_ (Ci/Ca) was observed, suggesting that rates of carbon assimilation were not limited by increasing stomata resistance to CO_2_ uptake [84,89]. Similar results were observed in the soybean–*P. pachyrhizi* pathosystem where a reduced stomatal aperture, which should reduce CO_2_ influx, was instead accompanied with an increase in internal CO_2_ concentration in infected leaves [83]. This raises the question if the reduction in assimilation and photosynthetic rate in infected plants is due to an increase in stomatal resistance to CO_2_ influx or the impairment of the photosynthetic apparatus. As hypothesized by Bassanezi et al. [84] and Lopes & Berger [89] the decreased carbon assimilations, allied to a rise in stomatal resistance and increased intercellular concentration of CO_2_ during disease development, could be in part explained by mesophyll resistance to CO_2_ diffusion to carboxylation sites, or some biochemical limitation on CO_2_ fixation within the chloroplasts. In agreement with the results of chlorophyll *a* fluorescence imaging, transcriptomic analysis of the interaction between soybean and Asian soybean rust revealed the down-regulation of genes encoding for enzymatic photosynthetic machinery and carbon fixation metabolism, such as chlorophyll *a*/*b* binding proteins, photosystem I reaction center, and photosystems II proteins [46,91].

### 3.2. Regulation of Carbohydrate Allocation during Plant-Pathogen Interaction

Within the initial stages of pathogen infection, a myriad of defense reactions are tightly regulated allowing an efficient resistance response to the invaded host cell. Some of the well-studied cellular reactions have been described in previous sections of this review, including the rearrangement of cell wall structural proteins, the induced controlled collapse of invaded host cells, the activation of biosynthetic pathways resulting on the production of phytoalexins, cytoskeleton reorganization, or ROS production. Naturally, the activation and the precise spatial-temporal coordination of these events are highly energy demanding, and the carbohydrates reserves in infected cells are critical to fuel such processes [92]. However, the high demand for carbohydrates from infected tissues with reduced photosynthetic activity leads to a shift from an assimilatory to a carbohydrate-consuming state. In these cases, carbohydrates supply can be maintained with increased activity of carbohydrate cleaving enzymes, such as invertases, and other primary carbon metabolism pathways [88].

Increasing invertase activity has been observed in plant-pathogen interactions, resulting in the irreversible hydrolysis of sucrose into glucose and fructose [93]. However, in most cases, it has not been possible to discern between host or pathogen contribution to the increased activity [94]. In one of the few studies of invertase activity focusing on legume and biotrophic pathogen, Voegele et al. [95], provide evidence for a pathogen contribution for increasing invertase activity in infected tissues in *V. faba-U. fabae* pathosystem. The authors identified a fungal gene (Uf*INV1*), with sequence homology to invertases, highly expressed soon after pathogen penetration and throughout the intercellular hyphae. The localization of this invertase expression in both the intercellular hyphae and haustoria complex might support a dual role of these enzymes in the biotrophic interaction with the host plant. While the apoplastic hydrolysis of sucrose through the secreted fungal invertase in early infection structures promote the conversion of infected tissue from source to sink and limit the export of carbohydrates from the infected tissue, the increased activity of these enzymes in the extra-haustorial matrix allows for a supply of substrate for the fungi carbohydrates transporters [95]. One example of this is the *U. fabae* monosaccharide transporter, HeXtrose Transporter 1 protein (HXT1p) [96]. In an attempt to search for genes potentially involved in the nutrient uptake by the pathogen in the *V. faba*-*U. fabae* pathosystem, Voegele et al. [96] revealed an abundantly expressed *in planta* induced gene, HeXose Transporter 1 (*HXT1*) in the rust haustoria, with high similarities to a variety of hexose transporters from other fungi. Analysis of *HXT1* transcripts accumulation and observations from immunofluorescence microscopy targeting HXT1p suggests that this transporter is confined to the haustorial plasma membrane, with substrate specificity for D-glucose and D-fructose [96]. This was one of the first studies providing evidence that haustoria are directly involved in sugar uptake and that this activity is possibly confined to haustoria. In the pea-powdery mildew pathosystem, glucose was also proven to be the major energy source to be transferred from the plant cell to the pathogen, with increasing uptake rates comparing to sucrose and fructose [97]. These observations suggest that the extra-haustorial matrix is likely a major source of carbohydrates for the hexose transporter, most likely produced through the combined action of both fungal and plant invertase enzymes [93,95].

## 4. Effectors Secretion and Interaction with Host Immune Molecular Responses

The same structures fundamental for pathogens to sequester host cell nutrient content are also important to maintain the compatibility of the pathosystem. Haustoria, given their intimate contact with host cell content, constitute the most likely, and most significant, source of effectors delivered through the extra-haustorial membrane [98]. For the purpose of this review, effectors are defined as pathogen-derived molecules released by the pathogen into the host cell or the apoplast, with a major purpose to facilitate successful colonization and completion of their life cycle, whereas by means of altering the host’s metabolism for their benefit or suppression of plant’s defenses (effector-triggered susceptibility, ETS) [99]. Nevertheless, released effectors can be recognized and became targets of resistance proteins in the host cell. In these cases, effectors’ recognition is mostly mediated intracellularly by a class of R (resistance) proteins with interactive domains, mostly nucleotide-binding domains and leucine-rich repeats (NLR) proteins, triggering an additional battery of defense reaction against the pathogen, termed effector-triggered immunity (ETI) [12].

Genome and transcriptome sequencing analysis of *E. pisi, U. appendiculatus*, and *P. pachyrhizi* haustoria isolated from infected leaves of *M. truncatula*, bean, and soybean, respectively, resulted in the identification of hundreds of genes likely involved in important aspects of haustoria biology, biotrophy, and pathogenicity [15,97,98,99,100]. In these cases, the prediction and identification of candidate effectors from data generated in high-throughput omics approaches are particularly hindered by the lack of a known common sequence characteristic of effector proteins. Nevertheless, several authors have defined a set of specific criteria to ease the identification of putative effectors: N- terminal signal sequence for secretion, small size, enriched cysteine residues, increased expression during stages of the infection processes, and strongly expressed *in planta* [100].

**Figure 2:**
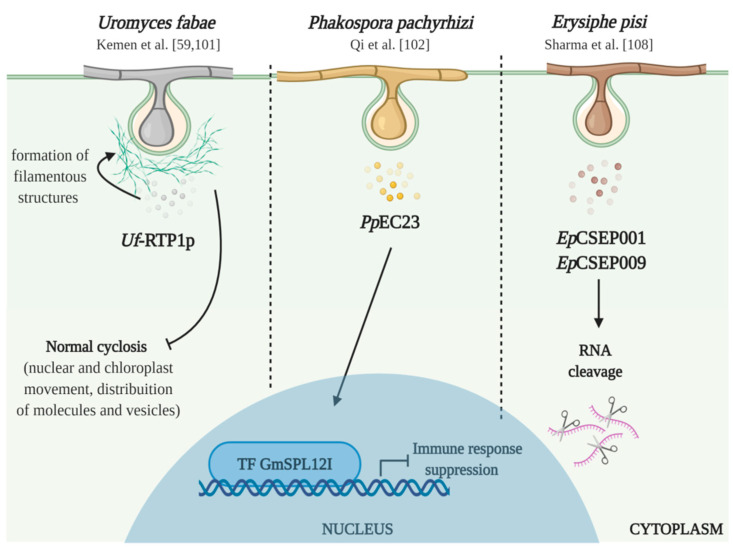
Effector proteins identified and characterized in legume crops. *Uf*-RTP1p released from *U. fabae* accumulates and forms filamentous like structures due to β-aggregation in the cytoplasm surrounding the haustorium. The *P. pachyrhizi* effector *Pp*EC23 effector interacts and possibly modulate post-translationally the soybean transcription factor *Gm*SPL12I, resulting in suppression of plant immunity. *Gm*SPL12I was shown to be a negative regulator of soybean defenses. Candidate secreted effectors *Ep*CSEP001 and *Ep*CSEP009 from *E. pisi* are analogous to RNase-like proteins and may express RNA cleavage activity (created with BioRender.com).

In legume-biotrophs pathosystems, the Rust Transferred Protein 1 (RTP1p) from *U. fabae* was one of the first effector proteins proven to be directly secreted through the extra-haustorial matrix and then transiting into the *Vicia faba* cytoplasm [59] (Figure 2).

Rust Transferred Protein 1 was also observed to aggregate and form filament-like structures within the cytoplasm of *V. fabae* cells and interfere with normal cytoplasmic streaming, thus potentially compromising the signal exchange within the host cell and the recruitment of molecules, organelles, and other components needed for the onset of defense mechanisms [101]. More direct observations of effector proteins influence on legume host immune system were reported by Qi et al. [102]. Among several *P. pachyrhizi* effector candidates, the authors detected one secreted cysteine-rich protein (*Pp*EC23) that interacts with soybean transcription factor (TF) *Gm*SPL12I (Figure 2). This TF was identified as a negative regulator of soybean immune response, has observed by the constitutive immunity expressed in *Gm*SPL12I-silenced soybean plants. In the particular case of *P. pachyrhizi*, one of the possible activities of the released effector is to manipulate the TF regulation or interfere with its functions through post-translational modifications, compromising plant immunity [102]. 

Given the proposed role of these secreted proteins for pathogenicity, as demonstrated by the works described above, one could hypothesize that silencing candidate fungal effectors could significantly impair pathogen colonization and disease development. In this context, host-induced gene silencing (HIGS) has been used to impair pathogenicity, through the production of host-derived small interfering RNAs (siRNA) that could induce RNA interference (RNAi) effects in haustorial-infected host cells. This approach has proven to be a valuable tool to assist the identification and characterization of novel candidate secreted effectors involved in pathogenicity in various cereal pathosystems: wheat—*Puccinia striiformis* [103], wheat—*P. triticina* [104], rice—*Magnaporthe oryzae* [105], barley and wheat infected with *Blumeria graminis* [106]. Silencing of five *U. appendiculatus* effector genes was obtained by using recombinant *Bean pod mottle virus* (BPMV) to transiently express antisense transcripts in common bean [107]. Virus-infected plants expressing siRNA for the candidate effectors developed fewer rust disease symptoms and accumulated less haustorium marker RNA, as compared to plants infected with BPMV not expressing gene fragments targeted for silencing. The results obtained seem to imply that siRNA present in the host cells is capable to trigger an RNA-mediated gene silencing of the corresponding gene released from the haustoria and compromise its pathogenicity [107]. To the best of our knowledge, this is the first example of a host-induced gene silencing approach applied to legume pathosystems. More recently, the infiltration of double-stranded RNA in pea leaves specifically designed to target a set of highly expressed candidate effector proteins identified in *E. pisi* haustorial transcriptome (*Ep*CSEP001 and *Ep*CSEP009), dramatically reduced disease symptoms compared to untreated control leaves [108]. Homology modeling of the selected candidate effectors revealed structure and sequence alignment similarities with ribonuclease (RNase)-like proteins and to the RNase-Like Proteins associated with Haustoria (RALPH) effectors’ family [108] (Figure 2). Effectors with RNase-like domains comprise the largest group of secreted candidate effectors in the genome of causal agent for cereal and grasses powdery mildew (*Blumeria graminis*) [109], further highlighting their crucial role during infection, in part modulated by the catalytic activity of specific host-derived RNA.

## 5. Genetic Basis of Resistance in Legume Crops against Biotrophs

Traditionally, research in plant disease resistance has mainly focused on the discovery and function of host immune components mostly encoded by dominantly inherited genes. The study of pathogens-derived effectors gave a significant contribution to precision breeding while using effectors as molecular probes to identify the corresponding R proteins in the host [110]. Other approaches have allowed to detect and characterized different types of resistance against biotrophic pathogens (Table 1).

However, the introgression of *R*-gene mediated resistance (often monogenic and inducing a complete or high level of resistance) into elite crops, leads to a risky scenario with high frequencies of resistance breakdown [9]. Additionally, given that efforts to introduce single or major genes are more amenable and less challenging, as compared to resistance controlled by several QTLs, lead to a current scenario in which most resistant varieties available are based on single genes [6]. Alternative strategies are available for breeders to develop more stable and durable resistance. Examples are the pyramiding of *R* genes into single genetic background, employment of recessive *R* gene, use resistance mechanisms of polygenic nature, or, the use of a mixture of cultivars expressing different resistance genes within one field ensuring the genetic diversity of agro-ecosystems, among others [9,108]. Adding to this topic, McDonald [13] points to the relevance of combining the strategies mentioned above to a dynamic turnover and diversity of resistance genes and resistant cultivars, regularly changed over time and space to contribute to highly effective and durable resistance.

Examples of single resistance genes are the already mentioned *er1*, *er2*, *Er3* genes conferring near complete resistant to *E. pisi* [25,40,111]. The particular example of *er1* constitutes the most widely deployed natural source of resistance in pea cultivars providing worldwide durable and broad-spectrum protection to *E. pisi* [127], However, *er1* and *Er3* have been proven to be ineffective against other powdery mildew pathogens infecting pea, as *E. trifolii* [128]. As for the complete resistance provided by *er2*, it seems to be more effective in some locations than others, which could be related to the existence of several races of *E. pisi* [129]. However the existence of *E. pisi* races with specific virulence it is yet to be described, to the best of our knowledge [39,129]. In pathosystems involving rust pathogens, most of the resistance described to date is incomplete, and often polygenic, as observed *in P. sativum-U. fabae* and *U. pisi* [34], chickpea—*U. ciceris-arietini* [130], lentil—*U. fabae* [33], among others. The term incomplete resistance if often applied to a host that is less affected by the pathogen, when compared to a susceptible control genotype, but without completely inhibit pathogen development and reproduction [7]. Nevertheless, several examples of nearly complete monogenic resistance (*Rpp1*, *Rpp2*, *Rpp3*) have been described in soybean to particular *P. pachyrhizi* isolates [121,131]. Given its race-specific nature, pyramiding of the mentioned resistance genes could be beneficial to confer a more stable and broader resistance to *P. pachyrhizi* [121]. Additional mapping studies have identified a number of QTLs conferring incomplete resistance to rust pathogens in other important legume crops, as summarized in Table 1.

## 6. Exploring Susceptibility Genes as an Alternative to *R*-Gene Based Breeding 

Susceptibility (*S*) genes encode for plant proteins that are targeted by pathogens to facilitate host colonization. As described before, often the released effectors by the adapted pathogen target key components of the plant’s immune system, in an attempt to subvert the onset of further defense responses [16,100]. However, effectors released can also interact and activate specific plant components, encoded by *S* genes, not necessarily involved with the plant’s immune system, that function as negative regulators of plant immunity by activating or stabilizing *S* genes and/or their products [127]. Therefore, the removal or inactivation of *S* genes could, in principle, impair the pathogen’s ability to cause disease and provide durable resistance. Indeed, to break *S* gene resistance, more than simply evading recognition by the host immune system components, the pathogen must overcome the dependency on a particular host factor needed for survival or to allow infection, which could imply the development of new strategies or functions by the pathogen, more difficult to achieve [132].

Characterization of the biological function of effector targets encoded by *S* genes and how they interact offers valuable knowledge as to how these host components can be exploited to induce a resistance phenotype. *Mildew locus O* (*MLO*) gene family, which encodes for a seven-transmembrane domain protein in plants, is perhaps one of the most recognized examples of susceptibility factors, impairing the response against powdery mildew infection [127]. Since its discovery in barley, several *MLO* homologs have been identified in the *MLO* susceptibility gene families of important plant species such as grapevine [133], tomato [134], wheat [135] and the model Arabidopsis [136,137]. 

Focusing on legumes, it was found that the function of the widely deployed *er1* resistance gene in pea was attributed to *MLO* loss-of-function mutation, resulting in recessively inherited and broad-spectrum resistance against *E. pisi*, expressed as the imposition of physical barriers to fungal penetration [138]. It appears that membrane rearrangement and regulation of vesicle trafficking involved in protein secretion at the plasma membrane are important factors for *mlo*-based resistance [139]. Restoration of susceptibility with transient expression of *P. sativum MLO* homologs in pea *mlo* mutants provided further evidence of the induced susceptibility caused by the *MLO* gene [138]. Despite phylogenetic distances, successful transgenic complementation of *mlo*-resistant pea genotypes by transgenic expression of *MLO* orthologs from other legume species, as observed in wheat and rice *mlo* orthologs [136], offers the opportunity to assess and confirm the function of other identified *MLO* genes across species [140,141].

Besides the *MLO* genes, there are some other examples of negative regulators of defense responses in legume species. In an attempt to better understand the role of MAP kinases (MPK) signaling pathways in the establishment of disease responses in soybean, Liu et al. [142] silenced the expression of genes encoding different MPKs using VIGS methodology. In this work, loss-of-function soybean mutants for the *GmMPK4* gene induce higher resistance to downy mildew (*Peronospora manschurica*) infection, detected as a strong reduction in mycelium epiphytic growth and penetration rate, as compared to vector control plants. The increased resistance of soybean mutant was attributed to an increase in salicylic acid and H_2_O_2_ accumulation, allied to the up-regulation of several genes involved in defense responses: lignin and phenylpropanoid biosynthesis processes, *Pathogenesis-related 2* gene (*Glyma19g31590*), SA and jasmonic acid (JA) signaling pathways. In Arabidopsis, the *P. syringae* effector AvrB was shown to enhance MPK4 phosphorylation, consequently promoting the kinase activity, suggesting that MPK could be targeted by the pathogen to facilitate colonization [143]. However, the potential application of *mpk4* in resistance breeding is compromised given the detrimental effects on other important phenotypic traits. Soybean plants with one of the two *MPK4* homologs (*MPK4A*) silenced induces severe symptoms, including stunted stature, tissue deformation, and early senescence [144,145].

Other studies in legumes highlighted the role that specific genes controlling the biosynthesis of the cuticle’s components could also be regarded as *S* genes. One example of this is the previously mentioned *IRG1* gene, which encodes for a TF controlling wax biosynthesis. In *M. truncatula*, loss-of-function mutation of *IRG1* resulted in a substantial decrease in wax primary alcohol groups and reduced surface hydrophobicity [27]. Such modifications in epicuticular wax composition induced a detrimental impact on the differentiation of pre-infection structures by the biotroph *P. pachyrhizi* [27].

The examples presented here clearly demonstrate that manipulation of *S* genes in legume crops offers a great potential for resistance breeding. One of the main obstacles to the exploitation of *S* genes while breeding for resistance comes from the fact that the existent *S* genes in plants frequently have an important biological function in developmental processes, with detrimental effects if mutated. However, research focused on the identification of *S* genes in legume species, and possible pleiotropic effects on other important traits resulting from *S* gene silencing is far behind when comparing to other important crops. It is imperative to expand our knowledge on this matter, to increase the collection of *S* gene identified in legume crops and the underlying mechanism, to fully understand if the use of *S* genes could induce a more durable, stable, and effective resistance against invading pathogens.

## 7. Concluding Remarks

This review has demonstrated that plants, in particular legume species, have evolved a complex multi-layered set of defense mechanisms (as briefly summarized in Table 2) tightly regulated inside the host cell, to prevent biotrophic colonization, that is presently, in the light of technological advances, being better understood.

Cell-wall appositions or papillae represent the first barrier against pathogen penetration. These structures are extremely complex and the accumulation of different molecules, including lignin, phenolic compounds, and ROS, determines the effectiveness of this physical and chemical barrier. Callose is the most abundant in these cell wall appositions and was proven crucial for the establishment of effective papillae. A deep understanding of the compositions of legume papillae, and what components will dictate a proper physical barrier, and the regulatory mechanisms of papillae formation, might result in new molecular approaches while breeding for increased penetration resistance. 

In direct association with the onset of cell wall reinforcements are the rapid reorganization and reorientation of microtubules and microfilaments at the host cell cytoskeleton. In fact, Arabidopsis mutants with disrupted trafficking of microfilament/microtubules prevented organelle aggregation and limit callose and lignin accumulations at the penetration site [146]. In legume species, treatments with actin polymerization inhibitors have also shown the importance of the cytoskeleton reorganization in the establishment of physical barriers; however, the mechanisms that lead to the induced susceptibility needs to be studied in more detail. Given the central role of the dynamic rearrangement of the cytoskeleton for a concerted defense response in invaded host cells, one could expect that pathogens have evolved strategies to directly or indirectly alter the cytoplasmic streaming. One potential example of this is the release of effector protein RTP1p from rust fungi, shown to accumulate and form filament-like aggregates throughout the host cytoplasm and inhibiting normal cyclosis to some extent. The identification of other effectors with similar activity and a better understanding of their structure, mode of function, and the specific targets in the host cell would contribute to uncover the mechanism of obligate biotrophs to ease accommodation in the host tissues.

As discussed in this review, the oxidative burst is considered a hallmark of successful recognition and response to pathogen invasion. The resulting rapid and transient accumulation of ROS can function directly in the establishment of important defense mechanisms previously discussed. However, it also becomes evident that ROS have an additional signaling function and interacts with other signaling molecules, TFs, and phytohormones (such as JA, ABA, SA, and ethylene), mediating the onset of supplementary defense barriers, yet to be critically described and characterized in legume-biotrophs pathosystems [147]. Due to the variety of signaling functions attributed to ROS, efforts should be made to gain a better understanding of the signaling pathways activated in these defense responses. In the particular case of HR-induction, it is becoming clear that ROS do not act only as damaging agents through excessive oxidation of cellular components, instead, they take an active role in a concerted cellular response, to which ROS-recognition mechanism are to be identified. Also, a better understanding of the fine-tuned mechanisms necessary for strict control of ROS concentration (e.g., compartmentalization or detoxification through antioxidative enzyme activity) holds promising insights into the network of defense responses mediated by these molecules. Regarding the study of the impact on photosynthetic apparatus, the advances in this field have been relatively slow in legume crops challenged with biotrophic pathogens. As previously mentioned, most studies have focused on the responses in cereals and Arabidopsis. However, the diverse array of impacts on physiological components detected in those different pathosystems, and the characteristic signatures of plant diseases, makes it unreliable to extrapolate observations obtained in different plant families into legume crops, and reinforces the need to promote this type of research in legumes. 

More recently, the fascinating field of effectors biology has experienced significant progress in legume species. Effectoromics screens of biotrophic pathogens have identified an ever-growing number of candidate effectors with a crucial role in establishing a successful infection while interfering with host immune responses. Specific gene expression *in planta*, and sequencing of isolated haustorial transcriptome from infected leaf, has been used as a starting point to predict and identify candidate effectors, with the potential to reduce large sets of predicted effectors to a more amenable number for functional validation. This approach has allowed gaining a deeper insight into the molecular functions of these proteins deployed into the host cell. Although recent advances in this field exist, our understanding of the structural composition of candidate effectors, their expression patterns, and biochemical mechanisms by which they interact with target proteins to modulate host immune response, remains limited in legumes. 

Crucial for the functional characterization of the predicted candidate secreted effectors is the development and availability of efficient transformation systems enabling genetic manipulation of biotrophic pathogens, yet to be widely available for the most damaging legume pathogens [99]. Nevertheless, promising results were obtained with the disruption of an effector gene in the oomycete *Phytophthora sojae* through the establishment of a CRISPR-Cas9 system [148]. Alternatively, the involvement of promising candidate effectors in pathogenicity is most often validated by HIGS as an RNA-interference based approach. Encouraging results obtained while adopting these gene silencing platforms further supports the idea that plant resistance can be achieved while targeting effectors’ functions. 

Complementary to the study of effectors biology and its importance in pathogenicity is the identification of host proteins specifically targeted by the released effectors, as factors inducing plant susceptibility. A well-known example of this is the *MLO* gene family, of which loss-of-function alleles were shown to confer broad-spectrum and durable powdery mildew resistance in pea [138]. Increasing efforts should be directed to study the negative regulators of defense responses to take full advantage of S genes, while at the same time, accounting for potential pleiotropic effects rising from their repression in host legumes.

Ultimately, a detailed understanding and further in-depth research of the cellular, molecular, and genetic components of the vast array of defense mechanisms is crucial for the development of efficient breeding initiatives for biotroph pathogen resistance. The intricate, highly complex, and heterogenic nature of the immune responses observed makes it particularly difficult to transfer the knowledge acquired from other non-legume species (e.g., cereals, Arabidopsis) to legume crops. Thus, an increasing effort should be directed to a thorough and detailed research on the biology of legumes interaction with pathogens. The techniques for probing complex biological systems are continuously expanding, providing unique data on multiple phenotypic layers as well as multiple ‘omics layers (genome, proteome, metabolome, among others). We need now to redirect these techniques to the legumes-biotrophs interaction investing in a comprehensive data analysis and integration. Only in this way, it will be possible to capture and fully understand the complexity of this biological system.

## Figures and Tables

**Table 1:** List of resistance genes/QTLs identified in important legumes against biotrophic pathogens.

Legume Species	Pathogen	Genetic Basis of Resistance	Resistance Gene/QTLs
*M. truncatula*	*E. pisi*	Polygenic	*Epp1* (LG4), *Epa1* and *Epa2* (LG5) [51]
*P. sativum*	*E. pisi*	Single recessive gene	*er1* (LG6), *er2* (LG3) [25]
*P. fulvum*	*E. pisi*	Single dominant gene	*Er3* (LG4) [111]
*L. cicera*	*E. pisi*	Polygenic	*EpDSI* (LGI), *EpDSII* (LGII), *EpDSIV* (LGIV)
*L. cicera*	*E. trifolii*	Polygenic	*EtDVIII* (LGVIII) [42]
*V. faba*	*U. fabae*	Single dominant gene	*Uvf-1* [112]
*Arachis hypogaea*	*Puccinia arachidis*	Polygenic	QTL_RUST_01-QTL_RUST_12 (LG1,2,3,6,7,8,9,10) [113] (3)
*C. arietinum x C. reticulatum*	*U. ciceris-arietini*	Polygenic	*Uca1/uca1* (LG7) [36]
*P.* vulgaris	*U. appendiculatus*	Single resistance gene	*Ur-3* (LG11) [114]*, Ur-4* (LG6) [115]*, Ur-5* (LG4) [116]*, Ur-6* (LG11) [117]*, Ur-7* (LG11) [118]*, Ur-9* (LG1)*, Ur-11* (LG11) [119]*, Ur-13* (LGB8) [120]
*G. max*	*P. pachyrhizi*	Single dominant gene	*Rpp1* (LG-G) [121]*, Rpp2* (LG-J) [122]*, Rpp3* (LG-C2) [123], *Rpp4 (LG-G)* [122], *Rpp5 (LG-*N) [124]
*Pisum sativum*	*U. fabae*	Single partially dominant gene	*Ruf* [125]
*P. fulvum*	*U. pisi*	Polygenic	*UpDSII* (LGII)*, UpDSIV* (LGIV)*, UpDSIV.2* (LGIV) [126]

**Table 2:** A summary of the main resistance mechanisms discussed in this review.

Resistance Mechanism	Molecular Components Potentially Involved	Pathosystem	References
**Inhibition of spores germination and differentiation of pre-infection structures**			
Epicuticular morphology; wax content	*IRG1*	*M. truncatula-P. pachyrhizi*	Ishiga et al. [26] Uppalapati et al. [27]
**Physical barriers to pathogen penetration**			
Callose, lignin deposition	*GSL5*	*V. faba-Pst*	Cheng et al. [30]
	Cellulose synthase family genes	*L. cicero-E. pisi*	Santos et al. [42]
	*GmPAL; GmO-MT*	Soybean*-P. pachyrhizi*	Cooper et al. [49] Pandey et al. [48]
Cell wall protein cross-linking	*er1*	Pea*-E. pisi*	Iglesias et al. [40]
**Post-penetration resistance**			
Hypersensitive response	*er2; Er3*	Pea-*E. pisi*	Chen & Heath [60]
	EDS1 pathway	*M. truncatula-E. pisi*	Foster-Hartnett et al. [53]
	Cinnamyl alcohol dehydrogenase	*V. faba*-*U. fabae*	Rojas-Molina et al. [55]
